# Prevalence of Sensitization to Panallergens and IgG4 Profiles Against Specific Foods in Patients with Allergic-Phenotype Eosinophilic Esophagitis

**DOI:** 10.3390/jcm15051728

**Published:** 2026-02-25

**Authors:** Joan Domenech Witek, Rosario González Mendiola, Margarita Tomás Pérez, Ambrosia A. Vásquez Bautista, Vicente Jover Cerdá, Clara Carballas Vázquez, Miguel Ángel Echenagusia Abendibar, María de los Ángeles Gonzalez Labrado, Inmaculada Ibarra Calabui, Raquel de la Varga Martinez, Jorge Mannelli Rius, Diego Gutiérrez Fernández

**Affiliations:** 1 Hospital Universitario Puerta del Mar, 11009 Cádiz, Spain; raqueldelavarga@hotmail.com (R.d.l.V.M.); jorgemannelli@icloud.com (J.M.R.); drgutifer60@gmail.com (D.G.F.); 2 Hospital Central de la Cruz Roja, 28003 Madrid, Spain; charogm2004@yahoo.es; 3 Hospital La Paz, 28046 Madrid, Spain; margui.tomas@gmail.com; 4 Hospital Universitario Puerta de Hierro, 28222 Madrid, Spain; angelalisa@hotmail.com; 5 Hospital General Elda, 03600 Alicante, Spain; algena00@hotmail.com; 6 Complejo Hospitalario Universitario, 15006 A Coruña, Spain; clara.carballas.vazquez@sergas.es; 7 Hospital de Mendaro, 20850 Mendaro, Spain; maetxenagusia@gmail.com; 8 Hospital Universitario Fundación Alcorcón, 28922 Madrid, Spain; mariagonzalezlabra@gmail.com; 9 Hospital Universitario Santa María del Rosell, 30203 Cartagena, Spain; adaibarracalabuig@gmail.com

**Keywords:** eosinophilic esophagitis, food allergy, IgG4, EDN

## Abstract

**Background:** The pathophysiological mechanism of eosinophilic esophagitis (EoE) is complex and is still being investigated. We believe that there is a group of patients with eosinophilic esophagitis which could be differentiated as having an allergic phenotype who exhibit a sensitization profile (aeroallergens, panallergens, foods and specific IgG4 levels) with significant differences compared to patients with conventional allergic disease without associated eosinophilic esophagitis and healthy controls. **Method:** We measured the prevalence of sensitization to aeroallergens, foods and panallergens by means of molecular diagnostic techniques (ImmunoCAP^TM^ ISAC) and determined the levels of specific IgG4 against foods and eosinophilic-derived neurotoxin (EDN) (ImmunoCAP technology) in patients with EoE of an allergic phenotype to study whether there are statistically significant differences with respect to the control groups (patients with different allergic pathologies without EoE and healthy patients without documented allergies). The total number of patients under study was 118, distributed among the different study groups. The case group (Allergic phenotype EoE patients) had 48 subjects. The food and respiratory allergy control groups had 30 subjects each. Finally, we included 10 in the healthy control group. **Results:** We were able to identify statistically significant differences when comparing levels of food-specific IgG4. Milk, egg, wheat, nuts, soy, cod, and Pru p3/LTP stood out. We did not observe significant differences in relation to sensitization to aeroallergens, foods, or panallergens. We also did not observe differences in EDN levels. **Conclusions:** We present a study in which statistically significant differences in IgG4 levels were observed in response to different types of food, comparing patients with eosinophilic esophagitis of allergic phenotype (case group) against subjects with allergic pathology without EoE and healthy subjects (control groups). Determining whether the detected foods are clinically relevant or not in these patients would be fundamental to establishing their usefulness as a treatment alternative in our patients.

## 1. Introduction

Eosinophilic esophagitis (EoE) is an inflammatory process that affects the esophageal mucosa and deeper layers of the esophagus. Its pathophysiological mechanism is complex and continues to be studied. It appears to be a mixed immune-mediated response to antigens and is not related to IgE-mediated mechanisms [1,2]. A multidisciplinary approach to studying this disease is essential. The clinical and research work carried out by specialists in gastroenterology, allergists, and/or pediatricians, as well as other specialists in this disease, has enabled progress in important areas such as epidemiology, diagnosis and treatment [3,4].

Although current guidelines have dismissed the use of classic allergy tests (IgE-mediated) to define certain treatments such as diets, their use plays an important role in the phenotyping of these patients, reporting a nearly 80% prevalence of sensitization to foods and/or aeroallergens in patients with EoE [5,6]. Distinct histopathological patterns (inflammatory, stenotic and mixed), which are not mutually exclusive, have been described [7,8].

In recent years, diagnosis in allergology has been progressing considerably. Currently, in routine clinical practice, we employ advanced techniques that enable precise diagnosis. Molecular diagnostics (ImmunoCAP^TM^ ISAC) has been particularly helpful in the field of food allergy and immunotherapy. For example, the detection of allergy to panallergens (antigens present in taxonomically related or unrelated species) explains numerous cross-reactivity phenomena between foods and aeroallergens. These phenomena and sensitization to panallergens may be relevant in patients with allergic-phenotype EoE [9,10,11]. It is very important to highlight the high level of sensitization observed in patients with EOE and the simultaneous presence of Th2 profile pathologies.

The role of IgG4 antibody responses in physiological and pathological settings has to be studied. The anti-inflammatory nature of IgG4 has been associated with dampening ongoing immune responses, but it is not clear if it can also cause pathology. The first steps towards understanding the pathological mechanisms underlying these IgG4-associated diseases have been taken, but little is still known regarding what triggers and maintains these IgG4 responses [12]. Although the use of specific IgG4 in the diagnosis of common food allergy has been dismissed, there are publications showing statistically significant differences in specific IgG4 levels of this subtype when comparing patients with eosinophilic esophagitis to healthy individuals [13,14]. The problem is that the few publications to date have studied a small number of patients and do not include control groups with allergic patients without EoE. We think that this could be a useful non-invasive diagnostic tool in EOE patients. In this regard, and as with other conditions such as asthma, finding useful and non-invasive tools for monitoring patients with EoE is essential. Biomarkers such as eosinophil cationic protein (ECP) have proven useful [15], and others like eosinophil-derived neurotoxin (EDN) could also be helpful in these patients. In fact, the EDN has been described as useful in asthmatic patients [16].

We believe that there is a group of patients with eosinophilic esophagitis which we could differentiate as having an allergic phenotype, and who exhibit a sensitization profile (aeroallergens, panallergens, foods and specific IgG4 levels) with significant differences when compared to patients with conventional allergic disease without associated eosinophilic esophagitis and healthy controls.

### Objectives

Main

To determine the prevalence of sensitization to aeroallergens, foods, and panallergens (LTPs, TLPs, PR10, profilin and tropomiosin) using molecular diagnostic techniques (ImmunoCAP^TM^ ISAC) and to measure levels of food-specific IgG4 (ImmunoCAP technology) in patients with allergic-phenotype EoE and to assess whether there are statistically significant differences compared with control groups (patients with exclusively IgE-mediated immediate food allergy without EoE, patients with respiratory allergy without EoE, and healthy individuals with no documented allergies).

Secondary

1. To investigate whether there is a correlation between sensitization to panallergens/aeroallergens and seasonal exacerbations in patients with allergic-phenotype EoE.
2. To investigate whether there are statistically significant differences in EDN levels among the different study groups.

## 2. Materials and Methods

### 2.1. Study Design

An observational, prospective, case–control, multicenter study. The study was reviewed and approved by the ethics committee of the Elda general hospital (Alicante, Spain).

### 2.2. Sample Selection

Patient selection was carried out during routine office visits. A total of 118 patients were selected (from all participating centers) for groups 1 (cases), 2, 3, and 4 (controls).

Group 1: Patients with allergic-phenotype EoE (EoE objectively diagnosed according to clinical criteria dysphagia, chest tightness, food impactions, or other compatible symptoms and the histological criterion of >15 eosinophils per high-power field in a biopsy from at least one esophageal segment, along with at least one allergic condition: rhinitis, asthma, and/or atopic dermatitis), who are either not receiving any pharmacological treatment or are on proton pump inhibitors (PPIs) and/or inhaled-swallowed corticosteroids, but who are not following a food elimination diet for their EoE (at least during the 12 weeks or 3 months prior to the study).

Group 2: Patients with IgE-mediated food allergy (type I mechanism). That is, patients who experience immediate symptoms, between 0 and 120 min after ingesting the food, with a clinical spectrum ranging from oral allergy syndrome to anaphylaxis. They may have other allergic diseases (rhinitis, asthma, or atopic dermatitis), but not EoE.

Group 3: Patients with respiratory allergy but no food allergy and no EoE. These patients did not report any symptoms suggestive of allergy related to food intake.

Group 4: 10 healthy control patients with no documented allergies of any kind.

Patients under the age of 18, with hypereosinophilic syndrome, hematological disorders, immunodeficiencies, or oncological processes, undergoing treatment with immunosuppressants or biological therapies, or EoE patients with food elimination diets or following them in the 12 weeks or 3 months prior to the study and EoE patients who are undergoing allergen immunotherapy were excluded.

### 2.3. Sampling

For convenience, based on the selection criteria, the sample size (*n*) was 118 patients taken from the various participating centers, and distributed among the different study groups. Forty-eight EoE patients with aeroallergens and food allergic sensitization, 30 patients with IgE mediated food allergy, 30 patients with respiratory allergy without any food allergy or food-related symptoms, and 10 healthy controls were included.

### 2.4. Sample Size

For an odds ratio of 4 or higher, it has been calculated that a sample size of 30 to 40 patients per group is sufficient (95% confidence level/80% statistical power).

### 2.5. Sample Processing

The samples were sent from the different participating centers to the reference laboratory. Serum samples were aliquoted upon receipt and stored at <−20 °C until analysis. Serum specific IgE, total IgE, and EDN concentrations were quantified using ImmunoCAP on a Phadia™ 250 platform (Thermo Fisher Scientific, Uppsala, Sweden). The EDN immunoassay employs immobilized anti-EDN antibodies for antigen capture and enzyme-labeled anti-EDN antibodies for fluorometric detection. Fluorescence response was proportional to EDN concentration and converted using a 2–200 µg/L calibration curve.

### 2.6. Statistical Study

Descriptive analysis, by variable type. Proportion for the qualitative variable, and measures of central tendency (mean, mode, and median) and dispersion (standard deviation, standard deviation, and variance) for the quantitative variable. For the most relevant variables, 95% confidence intervals were calculated.

In bivariate analysis, if the variable follows a normal distribution, parametric tests were used: chi-square to compare proportions and Student’s *t*-test to compare means. If the variable does not follow a normal distribution, nonparametric tests were used depending on the type of variable. Statistical significance was set at *p* < 0.05.

Multivariate analysis was used to minimize confounding bias; depending on the dependent variable, multivariate analysis was performed using stepwise binary logistic regression. As a measure of association, odds ratios were calculated with their 95% confidence intervals. To assess the multivariate model’s discriminatory ability, ROC curves were calculated along with their areas under the curve and 95% confidence intervals.

As this is a case–control study, we performed a binary multiple logistic regression analysis, expecting to obtain consistent crude and adjusted results. Three case–control studies were conducted: 1st, group 1 (cases) versus group 2 (controls with typical food allergy; 2nd, group 1 (cases) versus group 3 (controls with enviromental allergy) and 3rd, group 1 (cases) versus group 4 (health controls).

ROC curves were constructed based on the probabilities estimated by each logistic regression model. These curves represented the relationship between sensitivity (true positives/vertical axis) and 1-specificity (false positives/horizontal axis) for different model cut-off points. The 95% CIs of the AUC and the optimal cutoff point were calculated using Youden’s index (sensitivity + specificity − 1). The data used to construct the curves were the IgG4 levels corresponding to the foods in which statistically significant differences were observed.

## 3. Results

Finally, a total of 118 subjects were enrolled in the study, and their distribution across the different groups is shown in Table 1. Sociodemographic characteristics in terms of age and sex are described in Table 2 and Table 3. The age and sex distribution in the case/EoE group is consistent with previous reports [14].

Table 4, Table 5 and Table 6 show the IgE-mediated sensitization profiles of the different groups for foods, aeroallergens and panallergens. Table 7 shows the levels of specific IgG4 against the different foods tested.

The EoE group exhibited significantly higher levels of food-specific IgG4 to several foods, including wheat, peanut, soy, casein, cod, hazelnut, and almond, compared with control group 2 (Table 8). Compared to control group 3, there is a greater response in the EoE patient group for egg white, wheat, peanut, soy, casein, cod, walnut, hazelnut, almond, and Pru p3/LTP. In the case of cod, although there are significant differences, the effect size is small (0.020) (Table 9). The EDN levels observed were Group 1 56.59 ng/mL ± 18.30; Group 2 60.19 ng/mL ± 19.81; Group 3 47.34 ng/mL ± 18.21; Group 4 30.5 ng/mL ± 17.45. In both cases, no significant differences were observed in EDN levels or in sensitization to panallergens. No significant differences were observed in food and aeroallergen sensitization when comparing group 1 with groups 2 and 3.

Comparing the case group with the control group of healthy subjects, there is a greater response in the allergic EoE patient group compared to group 4 for egg white, wheat, soy, casein, cod, walnut, hazelnut, Phl p12, Pru p3, and Pen a1. In the case of Pen a1, although there are significant differences, the effect size is small (0.010) (Table 10).

**Figure 1:**
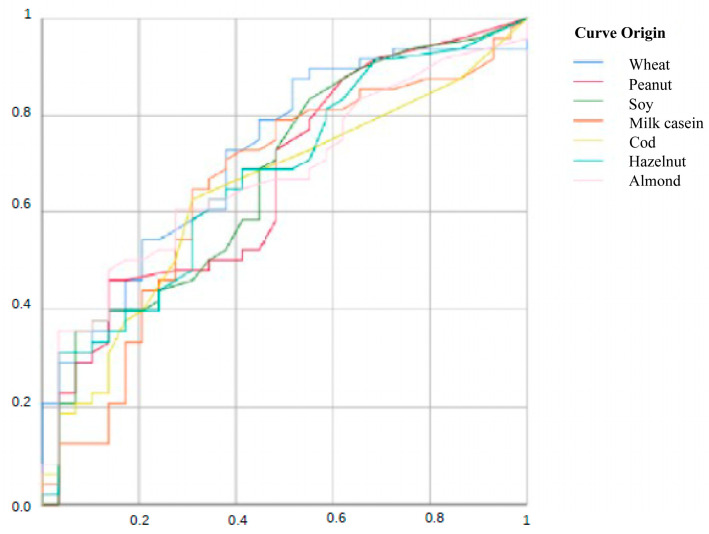
ROC Curve. Comparison of IgG4 levels between groups 1 and 2. Vertical axis: **sensitivity**; Horizontal axis: **specificity**.

Comparing groups 1 and 2, all foods show AUC (Figure 1) significantly > 0.5, indicating real discriminative capacity between allergic EoE and controls according to the IgG4 level. AUC values between 0.65 and 0.71 reflect moderate precision, useful as a complementary (not sole diagnostic) tool. On the other hand, values less than 0.65 indicate modest discrimination between groups.

**Figure 2:**
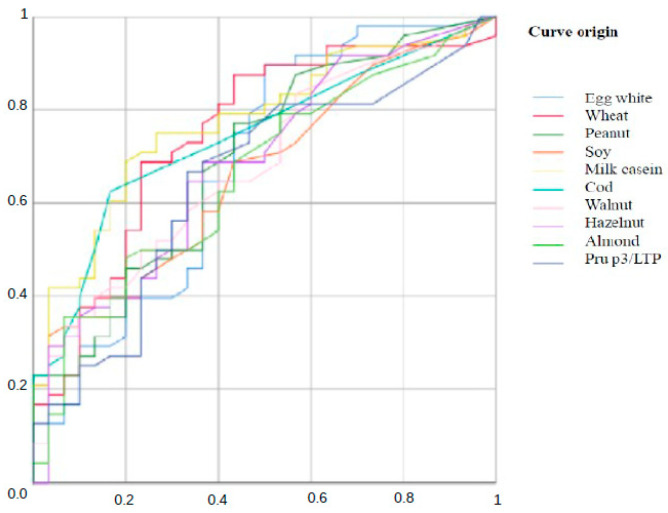
ROC Curve. Comparison of IgG4 levels between groups 1 and 3. Vertical axis: **sensitivity**; Horizontal axis: **specificity**.

Comparing groups 1 and 3, all foods show AUC (Figure 2) significantly > 0.5, indicating real discriminative capacity between allergic EoE and controls according to the IgG4 level. AUC values between 0.65 and 0.71 reflect moderate precision, useful as a complementary (not sole diagnostic) tool. On the other hand, values less than 0.65 indicate modest discrimination between groups.

The cutoff points for IgG4 levels observed when comparing groups 1 and 2 and groups 1 and 3 are shown in Table 11 and Table 12. The Youden’s index is a summary measure used in ROC curves to determine the optimal cutoff point of a biomarker, maximizing the sum of sensitivity and specificity. The values observed indicate moderate diagnostic performance (range 0 to 1), representing an acceptable ability to differentiate between cases and control individuals, with a moderate vertical distance between the ROC curve and the random line.

It is important to note that IMMUNOCAP ISAC ^®^ detected food allergy in 19 of the 48 patients in the EoE group (40%), whereas elevated IgG4 levels were observed in as many as 43 patients in this group (89%). Both techniques agreed on the detection of food allergy in 9 cases (21%), with wheat, egg white, and casein being the most commonly detected allergens. Although, in general, patients did not exhibit elevated IgG4 levels against panallergens, such levels were observed against Pru p3/LTPs in 12 of the 48 patients with EoE (25% of cases).

## 4. Discussion

We present a study in which statistically significant differences in IgG4 levels against different foods were observed when comparing patients with allergic-phenotype eosinophilic esophagitis (case group) with subjects with allergic disease but without EoE and healthy individuals (control groups).

Although the initial hypothesis posited the possibility of detecting differences in EDN levels or in the prevalence of sensitization to panallergens, no statistically significant differences were observed in this regard. It was only observed that EDN levels were higher in patients with allergic disease compared to those without documented allergy. We also did not observe any statistically significant differences in sensitization to aeroallergens or foods when comparing the groups of subjects with allergic disease.

In previous studies, significantly elevated specific IgG4 levels had been observed in EoE patients in response to foods such as wheat/gluten, milk, or egg [13,17]. In our study, we confirmed these foods and also observed significantly elevated levels regarding other foods such as nuts, soy, cod, or Pru p3/LTP. It is worth noting that these statistically significant differences are observed not only when compared with healthy controls, but also with patients with general allergic disease and food allergy without associated EoE. We believe that it is particularly valuable to observe significant differences when comparing with patients with IgE-mediated food allergy, which confirms that the immune mechanism in patients with EoE behaves in a different way.

As mentioned in the introduction, phenotyping in patients with EoE is essential to tailor management to each individual case. With regard to this phenotyping, identifying allergenic triggers is very important in allergic/Th2-type EoE profiles. The problem is that the immune response underlying the pathophysiological mechanism in EoE is complex. Currently, clinical guidelines have ruled out the exclusive use of IgE-mediated techniques to identify clinically relevant foods in patients with EoE. Therefore, going beyond IgE when evaluating these patients is essential.

In the present study, we confirm what was mentioned in the introduction regarding the potential role of IgG4 in identifying triggers in our patients. Compared to previous studies, we offer a larger sample size (*n*), comparisons across different groups, and a broader panel of potential triggers under investigation. Given the previous literature [12], we believe that the role of IgG4 may be an immune response by the patient to counteract a pathological response to an antigen to which the patient is repeatedly exposed and which is responsible for the underlying EoE inflammation. The presence of IgG4 deposits in the esophageal mucosa observed in patients with EoE in which foods such as milk are relevant, reinforce this hypothesis [18]. However, further studies are needed to understand this mechanism.

Once it has been confirmed that there are statistically significant differences between the various groups, it is important to establish the actual clinical relevance of the foods in which a significantly higher level is observed in the course of patients with allergic-phenotype EoE. Furthermore, the fact that there is some agreement between elevated Ig4 levels in response to certain foods and the foods identified as potential triggers by in vivo and in vitro IgE-mediated techniques reinforces that we could use IgE and no IgE-mediated techniques when evaluating our patients.

## 5. Conclusions

Conducting prospective studies with a sufficient number of patients to determine whether the identified foods are clinically relevant or not, based on the suggested combination of techniques, is the natural next step following this study that has been carried out. The objective would be to determine if targeted diets avoiding foods that present specific IgG4 high levels could be indicated to achieve clinical improvement and anatomopathological resolution in our patients. Additional studies are essential to clarify whether specific diets can become a useful alternative in the management of our patients. Currently, there is no evidence to that effect.

## Figures and Tables

**Table 1:** Sample disposition.

	n	%
**Group 1/Cases: patients with allergic-phenotype EoE**	48	40.67
**Group 2: patients with IgE-mediated food allergy without EoE**	30	25.4
**Group 3: patients with respiratory allergy without food allergy or EoE**	30	25.4
**Grupo 4: healthy controls**	10	8.5
**Total subjects**	118	100

**Table 2:** Sociodemographic characteristics: sex.

	Group 1		Group 2		Group 3		Group 4		Total	
	n	%	n	%	n	%	n	%	n	%
**Men**	31	64.6	10	33.3	13	43.3	3	33.3	57	48.3
**Women**	17	35.4	20	66.7	17	56.7	7	66.7	61	51.7
**Total**	48	100	30	100	30	100	10	100	118	100

**Table 3:** Sociodemographic characteristics: age.

	n	Minimum	Pct25	Mean	Median	Pct75	Maximum	Standard Deviation
**Group 1**	48	17	30.3	37.8	35.0	46.0	66.0	12.5
**Group 2**	30	19	22.3	33.5	27.5	42.5	72.0	15.2
**Group 3**	30	20	31.0	41.6	42.0	50.0	65.0	12.5
**Group 4**	10	28	40.3	49.3	49.0	53.8	75.0	14.2
**Total**	118	17	27.0	38.7	37.0	48.8	75.0	13.9

**Table 4:** IgE mediated food sensitization (ImmunoCAP^TM^ ISAC).

	Group 1		Group 2		Group 3		Group 4		Total	
	N	%	N	%	N	%	N	%	N	%
No food sensitization	28	70.0	7	23.3	29	96.7	10	100.0	74	62.7
With food sensitization	12	30.0	23	76.7	1	3.3	0	0.0	44	37.3
Nuts	5	12.5	9	30.0					14	11.9
Fruits	10	25.0	9	30.0					19	16.1
Shellfish	1	2.5	4	13.3					5	4.2
Milk	4	10.0	3	10.0					7	5.9
Egg	4	10.0	5	16.7	1	3.3			10	8.5
Fish	4	10.0	0	0.0					4	3.4
Wheat	4	10.0	5	16.7					9	7.6
Soy	2	5.0	7	23.3					9	7.6
Mussel	1	2.5	1	3.3					2	1.7
Cephalopods	1	2.5	1	3.3					2	1.7
Total	40	100	30	100	30	100	10	100	118	100

**Table 5:** IgE-mediated aeroallergen sensitization (ImmunoCAP^TM^ ISAC).

	Group 1		Group 2		Group 3		Group 4		Total	
	N	%	N	%	N	%	N	%	N	%
No aeroallergen sensitization	2	5.0	1	3.3	0	0.0	6	20.0	9	7.6
With aeroallergen sensitization	38	95.0	29	96.7	30	100	4	97.5	109	92.4
Grasses	36	94.7	17	58.6	14	46.7	1	25.0	68	62.4
*Olea europea*	22	57.9	11	37.9	12	40.0	1	25.0	46	42.2
*Salsola kali*	8	21.1	1	3.4	5	16.7	0	0.0	14	12.8
*Artemisia vulgaris*	6	15.8	5	17.2	4	13.3	0	0.0	15	13.8
*Cupressus arizonica*	24	63.2	10	34.5	19	63.3	1	25.0	54	49.5
Betula	2	5.3	6	20.7	2	6.7	0	0.0	10	9.2
*D. pteronyssinus*	17	44.7	8	27.6	5	16.7	0	0.0	30	27.5
*D. farinae*	17	44.7	7	24.1	3	10.0	0	0.0	27	24.8
Dog epithelium	15	39.5	12	41.4	15	50.0	0	0.0	42	38.5
Cat epithelium	22	57.9	14	48.3	8	26.7	0	0.0	44	40.4
*Alternaria alternata*	12	31.6	7	24.1	4	13.3	1	25.0	24	22.0
Plantago	5	13.2	3	10.3	2	6.7	0	0.0	10	9.2
*Platanus acerofila*	2	5.3	0	0.0	2	6.7	0	0.0	4	3.7
*Parietaria judaica*	1	2.6	0	0.0	1	3.3	0	0.0	2	1.8
Total	40	100	30	100	30	100	10	100	118	100

**Table 6:** IgE-mediated panallergen sensitization (ImmunoCAP^TM^ ISAC).

	Group 1		Group 2		Group 3		Group 4		Total	
	N	%	N	%	N	%	N	%	N	%
No panallergen sensitization	19	47.5	8	26.7	19	63.3	10	100	56	47.5
Panallergen sensitization	21	52.5	22	73.3	11	36.7	0	0.0	62	52.5
Pru p3/LTP	16	76.2	13	59.1	3	27.3	0	0.0	32	51.6
TLP	10	47.6	2	9.1	2	18.2	0	0.0	14	22.6
Profilin	9	42.9	9	40.9	4	36.4	0	0.0	22	35.5
PR10	2	9.5	6	27.3	3	27.3	0	0.0	11	17.7
Tropomiosin	3	14.3	5	22.7	0	0.0	0	0.0	8	12.9
CCD	6	28.6	3	13.6	7	63.6	0	0.0	16	25.8
Total	40	100	30	100	30	100	10	100	118	100

**Table 7:** Specific IgG4 levels in groups 1, 2, 3 and 4.

Foods	Group 1. EoE	Group 2	Group 3	Group 4
Egg white	7.06 ± 8.8 *	4.7 ± 7.6	3.68 ± 5.56	1.77 ± 1.92
Wheat	5.8 ± 9.5	1.1 ± 1.7	1.39 ± 2.9	0.05 ± 1.78
Peanut	1.1 ± 1.8	0.64 ± 2.4	0.29 ± 0.49	0.82 ± 2.02
Soybean	0.55 ± 0.82	0.24 ± 0.7	0.12 ± 0.18	0.08 ± 0.12
Casein	9.4 ± 17.7	5.4 ± 12.9	0.91 ± 2.2	0.62 ± 1.33
Cod	0.35 ± 0.85	0.09 ± 0.3	0.02 ± 0.03	0.08 ± 0.00
Walnut	0.7 ± 1.14	0.48 ± 1.55	0.2 ± 0.42	0.23 ± 0.58
Hazelnut	0.97 ± 1.43	0.43 ± 1.21	0.43 ± 1.25	0.16 ± 0.26
Almond	1.55 ± 2.19	0.43 ± 0.86	0.63 ± 1.29	1.01 ± 1.75
Phl p12	0.08 ± 0.1	0.05 ± 0.07	0.06 ± 0.06	0.02 ± 0.02
Bet v1	0.02 ± 0.03	0.02 ± 0.05	0.03 ± 0.09	0.006 ± 0.0
Pru p3/LTP	0.95 ± 2.34	0.51 ± 1.65	0.19 ± 0.32	0.06 ± 0.06
Pen a1	0.04 ± 0.04	0.02 ± 0.03	0.02 ± 0.03	0.005 ± 0.0

* IgG4 levels are expressed in micrograms/milliliter (mcg/mL). IgG4 values in each group are expressed as mean with standard deviation for a 95% confidence interval.

**Table 8:** Differences between groups in EDN and IgG4 values (groups 1 and 2).

	*p*-Value *	Effect **	IC95%
EDN	0.494	−5.865	−22.630–10.510
Egg white	0.055	1.230	−0.020–3.680
Wheat	0.001	0.900	0.300–1.640
Peanut	0.015	0.100	0.020–0.320
Soyben	0.012	0.050	0.010–0.140
Casein	0.027	0.770	0.030–2.770
Cod	0.038	0.010	0.000–0.030
Walnut	0.055	0.060	0.000–0.280
Hazelnut	0.012	0.170	0.020–0.370
Almond	0.010	0.320	0.030–0.740
Phl p12	0.291	0.010	−0.010–0.030
Bet v1	0.113	0.000	0.000–0.010
Pru p3/LTP	0.057	0.050	0.000–0.140
Pen a1	0.214	0.000	0.000–0.020

* *p*-value calculated using the Mann–Whitney test for nonparametric samples, with a 95% confidence level. ** Hodges–Lehmann median difference for independent samples.

**Table 9:** Differences between groups in EDN and IgG4 values (groups 1 and 3).

	*p*-Value *	Effect **	IC95%
EDN	0.444	4.340	−9.370–18.070
Egg white	0.007	1.540	0.270–4.040
Wheat	0.000	0.970	0.410–1.660
Peanut	0.004	0.100	0.030–0.330
Soybean	0.015	0.050	0.010–0.160
Casein	0.000	1.375	0.490–4.320
Cod	0.000	0.020	0.010–0.040
Walnut	0.008	0.100	0.020–0.300
Hazelnut	0.007	0.185	0.030–0.370
Almond	0.017	0.215	0.010–0.720
Phl p12	0.714	0.000	−0.020–0.030
Bet v1	0.230	0.000	0.000–0.010
Pru p3/LTP	0.027	0.065	0.010–0.150
Pen a1	0.117	0.010	0.000–0.020

* *p*-value calculated using the Mann–Whitney test for nonparametric samples, with a 95% confidence level. ** Hodges–Lehmann median difference for independent samples.

**Table 10:** Differences between groups in EDN and IgG4 values (groups 1 and 4).

	*p*-Value *	Effect **	IC95%
EDN	0.387	7.525	−8.250–34.780
Egg white	0.032	2.300	0.130–6.520
Wheat	0.008	1.020	0.280–3.170
Peanut	0.065	0.100	−0.010–0.450
Soybean	0.010	0.060	0.010–0.410
Casein	0.003	1.580	0.200–6.650
Cod	0.001	0.030	0.010–0.060
Walnut	0.013	0.110	0.020–0.520
Hazelnut	0.007	0.260	0.030–0.850
Almond	0.703	0.050	−0.340–1.190
Phl p12	0.032	0.025	0.000–0.070
Bet v1	0.084	0.000	0.000–0.020
Pru p3/LTP	0.008	0.100	0.020–0.260
Pen a1	0.009	0.010	0.000–0.030

* *p*-value calculated using the Mann–Whitney test for nonparametric samples, with a 95% confidence level. ** Hodges–Lehmann median difference for independent samples.

**Table 11:** Cutoff points in the comparison of groups 1 and 2.

	Probability	Sensitivity	Specificity	Youden’s Index (J)
Wheat	0.365	0.875	0.483	0.358
Peanut	0.33	0.458	0.862	0.32
Soybean	0.295	0.354	0.931	0.285
Casein	0.79	0.646	0.69	0.336
Cod	0.025	0.625	0.69	0.315
Hazelnut	0.225	0.625	0.655	0.28
Almond	0.705	0.479	0.862	0.341

**Table 12:** Cutoff points in the comparison of groups 1 and 3.

	Probability	Sensitivity	Specificity	Youden’s index (J)
Egg white	0.285	0.896	0.5	0.396
Wheat	0.915	0.688	0.767	0.455
Peanut	0.08	0.771	0.567	0.338
Soybean	0.47	0.313	0.967	0.28
Casein	0.635	0.688	0.8	0.488
Cod	0.025	0.625	0.833	0.458
Walnut	0.195	0.5	0.767	0.267
Hazelnut	0.135	0.688	0.633	0.321

## Data Availability

Data supporting reported results can be asked to corresponding author by email: joanwitek@yahoo.es.

## Abbreviations

The following abbreviations are used in this manuscript:

EoE	eosinophilic esophagitis
EDN	eosinophil derived neurotoxin
ECP	Eosinophilic cationic protein
LTP	lipid transfer protein
IgE	immunoglobulin E
IgG4	immunoglobulin G4
PPI	proton pump inhibitors
TLP	Thaumatin-like proteins
